# Negative emotions affect postoperative scores for evaluating functional knee recovery and quality of life after total knee replacement

**DOI:** 10.1590/1414-431X20154616

**Published:** 2015-11-17

**Authors:** A. Qi, C. Lin, A. Zhou, J. Du, X. Jia, L. Sun, G. Zhang, L. Zhang, M. Liu

**Affiliations:** 1 Department of Nursing, Laiwu People’s Hospital, Laiwu, China; 2 Department of Cardiology, Laiwu People’s Hospital, Laiwu, China; 3 The First Ward of the Surgical Department, Laiwu People’s Hospital, Laiwu, China; 4 Department of Internal Medicine, Branch of Laiwu People's Hospital, Laiwu People’s Hospital, Laiwu, China; 5 Department of Traumatic Orthopedics, Shengjing Hospital, China Medical University, Shenyang, China

**Keywords:** Total knee replacement, Negative emotion, Anxiety, Depression, Functional recovery, Health-related quality of life

## Abstract

This study aimed to determine whether psychological factors affect health-related quality of life (HRQL) and recovery of knee function in total knee replacement (TKR) patients. A total of 119 TKR patients (male: 38; female: 81) completed the Beck Anxiety Inventory (BAI), Beck Depression Inventory (BDI), State Trait Anxiety Inventory (STAI), Eysenck Personality Questionnaire-revised (EPQR-S), Knee Society Score (KSS), and HRQL (SF-36). At 1 and 6 months after surgery, anxiety, depression, and KSS scores in TKR patients were significantly better compared with those preoperatively (P<0.05). SF-36 scores at the sixth month after surgery were significantly improved compared with preoperative scores (P<0.001). Preoperative Physical Component Summary Scale (PCS) and Mental Component Summary Scale (MCS) scores were negatively associated with extraversion (E score) (B=-0.986 and -0.967, respectively, both P<0.05). Postoperative PCS and State Anxiety Inventory (SAI) scores were negatively associated with neuroticism (N score; B=-0.137 and -0.991, respectively, both P<0.05). Postoperative MCS, SAI, Trait Anxiety Inventory (TAI), and BAI scores were also negatively associated with the N score (B=-0.367, -0.107, -0.281, and -0.851, respectively, all P<0.05). The KSS function score at the sixth month after surgery was negatively associated with TAI and N scores (B=-0.315 and -0.532, respectively, both P<0.05), but positively associated with the E score (B=0.215, P<0.05). The postoperative KSS joint score was positively associated with postoperative PCS (B=0.356, P<0.05). In conclusion, for TKR patients, the scores used for evaluating recovery of knee function and HRQL after 6 months are inversely associated with the presence of negative emotions.

## Introduction

Osteoarthritis (OA) is a debilitating degenerative joint disease that is characterized by underlying erosion of articular cartilage and subchondral bone (1). The knee joints are among the earliest and most frequent joints involved in OA. OA patients suffer from considerable functional impairment of the knee joint and severe chronic pain. Among the available treatments, total knee replacement (TKR) is the safest and most cost-effective method to alleviate pain and to restore function of the knee joint in OA patients (2,3). Despite these advantages, many TKR patients still have residual knee impairment and functional limitations as compared with age-matched controls. One year after TKR, patients walk 15% slower than age-matched healthy individuals with no known knee pathologies (4,5). Interestingly, previous studies have shown that complications associated with TKR are not due to failed surgical procedures but might be closely related to the patient’s emotional health or to other long-term or perioperative psychological factors (6,7).

Although TKR is a safe and highly successful procedure in OA patients, some patients suffer from persistent pain and exhibit only partial recovery after TKR (8). Many patients who choose surgery experience negative moods preoperatively, including anxiety, depression and fear, in anticipation of potential surgery-related complications or death, as well as anxiety about the postoperative recovery phase (9). A previous study reported that patients with distressed mental states postoperatively had a greater risk of continued physical disability (10). Furthermore, mental distress resulting from negative emotions is associated with greater difficulty during recovery after TKR, with sharper pain levels and greater limitations of joint function and mobility (11). Therefore, in a subset of patients, negative emotions increase the risk of poor outcomes, including decreased health-related quality of life (HRQL) and increased severity of physical disability and discomfort (12). Recurrent pain after surgery is a frequent, and sometimes severe, health problem that significantly affects the HRQL of OA patients (13). Accordingly, besides better preparing patients for the experience of surgery, potentially, a major improvement in outcomes could be achieved if patients could avoid preoperative and postoperative distress and depression (9). Therefore, the present study evaluated the effect of negative emotions, such as anxiety, depression, and personality factors, on HRQL and recovery of knee joint function in patients after TKR. We anticipate that this study will contribute to identify more effective ways to overcome the challenges encountered by patients during postoperative recovery and to accelerate their social re-adaptation (especially on a psychological level), thus improving their HRQL.

## Material and Methods

### Ethics statement

This study was performed after obtaining approval from the Institutional Review Board of Laiwu People’s Hospital. Informed written consent was obtained from each eligible participant, and the study was conducted according to the Declaration of Helsinki (14).

### Study subjects

A questionnaire-based survey was conducted on patients who underwent unilateral TKR in the Department of Surgery of Laiwu People’s Hospital between February 2013 and January 2014. The inclusion criteria for patients in this study were as follows: 1) patients who underwent TKR for OA, degenerative OA, or rheumatoid arthritis; 2) no history of knee surgery or hip surgery; 3) no psychiatric history; 4) written informed consent was available; and 5) all questionnaires were completed. A total of 135 eligible patients were enrolled in this study and were asked to complete extensive questionnaires upon admission within 1 week before surgery. After surgery, the same questionnaires were mailed to each patient at 1 and 6 months after surgery to assess short-term and long-term postoperative treatment effects, respectively. A total of 119 patients (38 men and 81 women; mean age, 62.1±10.12 years old) completed all of the required questionnaires to be eligible for inclusion in this study. The entire study was designed as a prospective trial with consecutive patients, and a comparative study was also performed based on the preoperative and postoperative treatment effects in the patients.

### Questionnaires

General information that was collected from the patients included age, gender, marital status, degree of education, average monthly income, occupation, and source of medical expenses. The following questionnaires were also conducted to collect study-related patient information. 1) The State-Trait Anxiety Inventory (STAI), which has a total of 40 items that are mainly used for evaluation of trait anxiety. The STAI includes a State Anxiety Inventory (SAI), which is used for evaluating short-term anxiety, and a Trait Anxiety Inventory (TAI), which is used for evaluating long-term anxiety. The maximum score on this questionnaire is 80 and the minimum score is 20 (15). 2) The Beck Anxiety Inventory (BAI), which is mainly used for the evaluation of anxiety states, includes 21 different anxiety symptoms. Participants must give the personal effects of each symptom score from 0 (no effect) to 3 (severe effects) (16). The Beck Depression Inventory (BDI) includes 21 self-assessments of depression symptoms, and each question has four self-assessment indices to reflect the degree of depression: score <11, no depression; score ranging from 11 to 15, potential depressive symptoms; score >16, depression; and score >25, severe depression (17). 3) The Eysenck Personality Questionnaire-Revised (EPQR-S) includes four parts as follows: extraversion or introversion (E); neuroticism (N); psychoticism (P), and lying and dissimulation (L). The standard T score=50+10×(original score of the participant - mean score of all participants)/standard deviation (18). 4) The Knee Society Score (KSS) includes a joint score (pain score of 50, activity range score of 25, and stability score of 25) and joint function score (climbing the stairs score of 50 and walking distance score of 50) (19,20). 5) The Short-Form Health Survey questionnaire (SF-36) (Chinese version) was used to evaluate the patients’ HRQL, which included body pain (BP), physical functioning (PF), physical role limitation (RP), emotional role limitation (RE), mental health (MH), social functioning (SF), vitality (VT), general health (GH), a Physical Component Summary Scale (PCS), and a Mental Component Summary Scale (MCS). The range in score for each part was 0-100, directly reflecting the participants’ health conditions (21). The patients’ psychological state and activity function 1 week before surgery were evaluated by the BAI, BDI, STAI, EPQR-S, KSS, and SF-36. The patients completed the BAI, BDI, KSS, and SF-36 questionnaires at 1 and 6 months postoperatively, and the scores of each scale were calculated.

### Statistical analysis

Statistical analysis was performed with the SPSS software, version 18.0 (USA). Data are reported as means±SD. The differences in scores (preoperatively and postoperatively) were evaluated using the paired *t*-test. The associations of psychological and psychic factors with HRQL and KSS scores were analyzed by performing multiple linear regression using the enter method, which simultaneously adds all of the variables to the model. Statistical results were considered significant when the P value was less than 0.05.

## Results

### Scale scores before and after surgery

The results of the STAI are reported in Table 1. The preoperative scores of the SAI and TAI were 44.52±10.12 and 43.61±9.23, respectively. The average scores were 49.39 (P score), 49.45 (E score), 55.68 (N score), and 48.11 (L score). The preoperative score for the BAI was 38.15±8.42. The BAI scores at the 1st month after surgery and at the sixth month after surgery were 32.16±6.48 and 28.69±7.52, respectively. BAI scores 1 month after surgery were significantly lower than preoperative scores (P<0.05). BAI scores 6 months after surgery were significantly lower than BAI scores 1 month after surgery (P<0.05). The preoperative BDI score was 10.25±4.26. BDI scores 1 and 6 months after surgery were 7.36±4.05 and 5.45±3.12, respectively. BDI scores 1 month after surgery were significantly lower than preoperative scores (P<0.05). BDI scores 6 months after surgery were significantly lower than BDI scores 1 month after surgery (P<0.05). KSS function scores 1 and 6 months after surgery were significantly improved compared with the preoperative scores (both P<0.05).

**Table:**
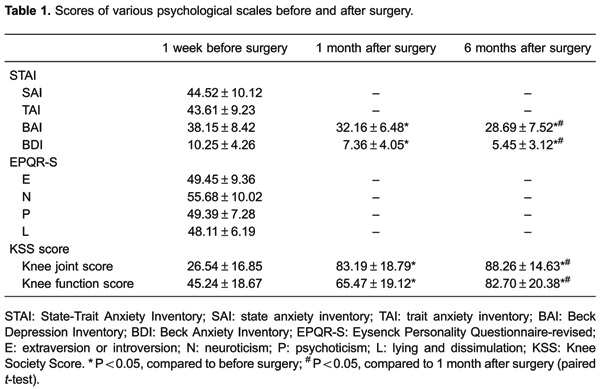

### Evaluation of HRQL

Table 2 shows the eight life aspects (BP, PF, RP, RE, MH, SF, VT, and GH) of the SF-36 score 6 months after surgery. The patients’ SF-36 scores 6 months after surgery in these eight aspects were significantly higher than preoperative scores (P<0.001). The PCS and MCS scores 6 months after surgery were higher than those preoperatively (P<0.001).

**Table:**
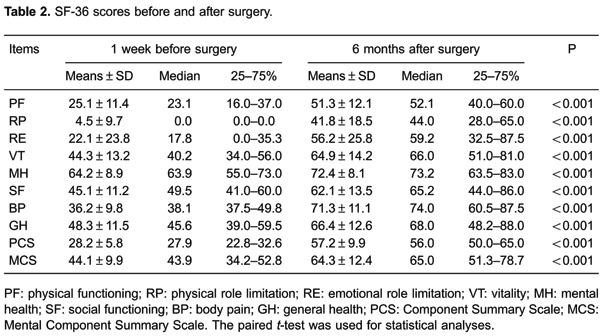

### Effects of psychological and psychic factors on HRQL

Multiple linear regression analysis was performed with PCS and MCS as dependent variables and SAI, TAI, BAI, BDI, P, E, N, and L as independent variables. Regression analysis showed that preoperative PCS and MCS scores were negatively associated with the E score (B=-0.986, B=-0.967, respectively, both P*<*0.05). Postoperative PCS and SAI scores were also negatively associated with the E score (B=-0.137, B=-0.991, respectively, both P<0.05). Postoperative MCS, SAI, TAI, and BAI scores were negatively associated with the N score (B=-0.367, B=-0.107, B=-0.281, B=-0.851, respectively, all P*<*0.05; Table 3).

### Associations of psychological and psychic factors with the KSS score

Multiple linear regression analysis was performed with knee joint and function scores 6 months after surgery as the dependent variables and SAI, TAI, BAI, BDI, P, E, N, L, PCS, and MCS as independent variables. Regression analysis showed that knee function 6 months after surgery was negatively associated with TAI and N scores (B=-0.315, B=-0.532, respectively, both P<0.05), but positively associated with the E score (B=0.215, P*<*0.05). The KSS joint score was positively associated with the postoperative PCS score (B=0.356, P<0.05; Table 4).

**Table:**
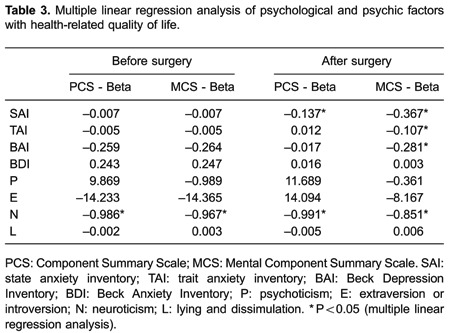

**Table:**
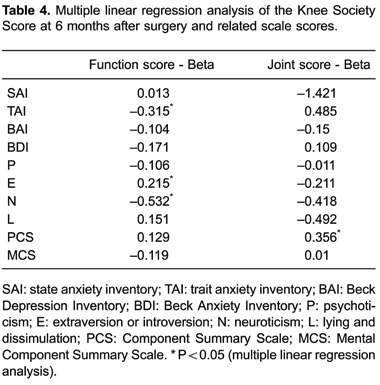

## Discussion

One of the main results of the present study was reduced strength of patients exhibiting negative emotions of depression and anxiety after TKR. Similar to patients with other diseases undergoing elective surgery, OA patients often experience high levels of anxiety before surgery because of the possibility of surgery-related complications or death. The use of surgical instruments and the thought of tissue penetration also often provoke fear and discomfort, and have been mentally associated with pain, destruction of body shape, and death (22). Furthermore, fear of neglect by society and family during the recovery phase, and patients’ inferiority complexes and annoyance with discomfort can also lead to negative emotions such as depression, anxiety, paranoia, and fear (23). TKR is a safe surgical option for OA patients, but only a small proportion of the patient population show complete postoperative improvement in physical function, pain, and quality of life (24,25). Previous studies have suggested that psychological factors contribute to poor outcomes after TKR (26,27). Our study suggested that patients who underwent TKR showed excellent recovery of knee function and improvement in HRQL, but the scores used for evaluating recovery of knee function and HRQL after 6 months were inversely associated with the presence of negative emotions. We demonstrated that HRQL in patients at 6 months after unilateral TKR was significantly improved. However, based on our results, improvement in the MCS was less than the observed improvement in the PCS. Although improvement in physical function is the main factor contributing to patients’ satisfaction with TKR, postoperative changes observed in the mental and emotional states are significantly different than the preoperative condition (28). A previous study showed that patients with preoperative anxiety or depression were less satisfied with TKR (29). Consequently, chronic pain or negative emotions in patients postoperatively might be a risk factor for decreased function rehabilitation, affecting the efficacy of TKR.

Negative emotions, including neurotic mood, anxiety, and depression, could affect improvement in HRQL. The present study showed that PCS and MCS scores were negatively associated with the neurotic value. This finding indicates that a lower level of HRQL might be associated with the personality traits of each patient. Patients with high neuroticism scores on the EPQ might have mental illnesses. High neuroticism is one of the risk factors for depression (30). Additionally, mental health affects TKR outcomes, and neurotic patients are at increased risk for physical disability after surgery because negative emotions have side effects that affect recovery of body function (10). Consequently, patients with anxiety and depression might require a longer rehabilitation time, which could affect their quality of life (31). Moreover, negative thought patterns, hypervigilance, fear, and avoidance could result in physical deterioration, based on the fear-avoidance model of chronic pain (9). Therefore, postoperative recovery and disease severity are negatively affected in patients with high negative emotion levels after TKR. The findings of this study are consistent with previous studies showing that high levels of anxiety and depression predict poorer quality of life and increase the severity of pain after surgery (11,32,33).

Our results strongly suggest that counseling of patients is urgently required to promote positive emotions and to improve outcomes. Satisfaction with in-hospital patient care could positively affect patients’ moods, and caregivers’ performance directly influences the quality of daily care (28). Therefore, daily care should be improved to enhance the positive emotions of patients. Additionally, postoperative pain is a major factor causing negative moods. Therefore, immediate and effective relief of postoperative pain might reduce anxiety and depression (34). Targeted and personalized rehabilitation, exercise, and sports programs might also help in improving knee function, mobility, and the moods of patients (8).

There are several limitations in this study. First, the number of patients in our study was small. Second, other variables, such as comorbidities or environmental factors, could have affected HRQL, but we failed to analyze these factors because of insufficient information. Finally, all of the enrolled patients were selected from a hospital setting in a specific geographical region. Therefore, the results might only be representative of this group of patients, and different results might be obtained in other geographical areas. However, this study provided important information about the factors affecting patients’ outcomes and satisfaction after TKR.

In conclusion, recovery of knee function and HRQL in OA patients is significantly improved after TKR. However, the postoperative scores used for evaluating recovery of knee function and HRQL are affected by negative emotions, including neuroticism, anxiety, and depression. The finding that negative mood has an inverse correlation with recovery of knee function and quality of life warrants further examination.
